# Patient Perspective on the Monitoring of Their Wet Age-Related Macular Degeneration during Coronavirus Disease 2019: A Retrospective Study

**DOI:** 10.3390/medicina59030490

**Published:** 2023-03-02

**Authors:** Georgios N. Tsiropoulos, Rodolphe Vallée, Coraline Calci, Daniela Gallo Castro, Aude Ambresin

**Affiliations:** 1 Swiss Visio Montchoisi, 1006 Lausanne, Switzerland; 2 RétinElysée, Ophthalmology Center, 1006 Lausanne, Switzerland; 3 Department of Health Sciences, Medical School, Aristotle University of Thessaloniki (A.U.Th), 54124 Thessaloniki, Greece; 4 Laboratory of Mathematics and Applications (LMA) CNRS UMR7348, Data Analysis and Computations through Imaging Modeling Mathematics (DACTIM-MIS Team) University of Poitiers, 15 Rue de l’Hôtel Dieu, TSA 71117, 86000 Poitiers, France; 5 Faculty of Biology and Medicine, University of Lausanne (UNIL), 1005 Lausanne, Switzerland

**Keywords:** anti-vascular endothelial growth factor, wet age-related macular degeneration, coronavirus disease 2019, anti-VEGF, wet AMD, COVID-19 patient perspective

## Abstract

*Background and Objectives*: The purpose was to provide the patients’ perspective on the monitoring of their wet age-related macular degeneration (wet AMD) during coronavirus disease 2019 (COVID-19) and the importance of telemedicine. *Materials and Methods*: Wet AMD patients that underwent intravitreal anti-vascular endothelial growth factor (anti-VEGF) injections in two Swiss ophthalmology clinics, completed two questionnaires after the first confinement due to COVID-19 in Switzerland. The first evaluated their views concerning their adherence to scheduled injections during the confinement, and the application of telemedicine in the future. The second, adapted from the National Eye Institute Visual Function Questionnaire-25, assessed their opinions on visual function change during confinement. *Results*: From a total of 130 patients, 8.5% responded they did not respect their assigned schedule (group 1) while 91.5% responded they did (group 2). A total of 78.7% of group 2 considered treatment reception as more relevant compared to the risk of COVID-19 contraction. During the pre-lockdown period, group 2 patients required more help from others than group 1 patients (*p* = 0.02). In the possibility of another lockdown, 36.3% of group 1 and 8.7% of group 2 would choose telemedicine to monitor their wet AMD (*p* = 0.02), 54.5% and 86.9% would rather visit the clinic (*p* = 0.02), while 9.0% and 4.3% would cancel their appointment, respectively. It was found that 70% of group 1 and 33.6% of group 2 would prefer to use the telemedicine services than visiting a telemedicine centre (*p* = 0.04). *Conclusions*: During circumstances similar to the COVID-19 confinement, most patients would prefer to visit the clinic. Group 1 would prefer wet AMD monitoring via telemedicine at a higher rate than group 2.

## 1. Introduction

In Western countries, age-related macular degeneration (AMD) is the main cause of vision loss in adults over the age of 65 years [1,2]. Dry AMD, which accounts for 90% of cases of AMD, is characterized by irreversible central vision loss because of the gradual apoptosis of the retinal pigment epithelium, neuroretina, and choriocapillaris and has no effective therapy [3]. Photoreceptor impairment due to pathological neovascularization characterises the exudative, also known as wet, form of AMD, or neovascular AMD (nAMD), which accounts for 10% of AMD cases [3]. The gold-standard treatment for wet AMD is intravitreal anti-vascular endothelial growth factor (anti-VEGF) injections [4]. The novel coronavirus disease 2019 (COVID-19), caused by severe acute respiratory syndrome coronavirus-2, has affected the treatment of wet AMD, as worldwide retinal expert committees replied to this new circumstance by decreasing the number of treatments that should be delivered to only those that are urgent or emergent [5,6]. Many elderly patients have missed a number of their elective visits for intravitreal anti-VEGF injections due to the COVID-19 restrictions and due to the fear of COVID-19 contraction [7].

Telemedicine is a new form of practice that allows healthcare workers to provide medical services remotely [8,9]. Despite the significant amount of research studies on telemedicine, there is a lack of high-quality evidence regarding its effectiveness, patient opinion, and cost [10].

Although telemedicine was used to monitor wet AMD before the beginning of COVID-19 [11], the need for digital personalised care became inevitable due to the pressure on the medical centre resources [12,13]. Telemedicine has assisted significantly in the screening, diagnosis, and progression monitoring of patients with AMD during COVID-19 [14]. Because the best-corrected visual acuity (BCVA) and optical coherence tomography (OCT) findings are the primary determinants of AMD treatment [11], telemedicine could be just as beneficial as face-to-face office consultations. OCT has the capability of detecting the location and type of fluid (intraretinal fluid, subretinal fluid, and pigment epithelial detachment) in patients with wet AMD, a fact of paramount importance as it is proven that BCVA is better in eyes with no intraretinal fluid (cysts or oedema) observed on optical coherence tomography than in eyes with persistent intraretinal fluid [15,16]. The presence of intraretinal fluid negatively affects BCVA more than the presence of subretinal fluid, though both have deteriorating effects on vision [15,16]. Pigment epithelial detachment is a critical biomarker for long-term vision loss in individualized anti-VEGF therapy, according to Schmidt-Erfurth and Waldstein [16]. The combination of these critical OCT findings with digital innovations [11], such as 5th generation wireless networks, artificial intelligence, machine learning, deep learning, and the internet of things, could ameliorate the monitoring of wet AMD patients, as the patients with threatening OCT features would be prioritized and would receive an intravitreal anti-VEGF injection immediately. On the other hand, patients in which the wet AMD is considered to be stable, will have more flexibility in arranging their next intravitreal anti-VEGF injection visit. This would ameliorate the patients’ quality of life and their attendance to their intravitreal anti-VEGF injection appointments. The approach of giving prioritized treatment to patients at risk of wet AMD progression, due to the screening via telemedicine, would secure a precise, high-level, individualized wet AMD management which is of vital importance, even more so during future confinement due to COVID-19 or any other similar circumstances. Given the fact that telemedicine seems to be the future in medical practice, ophthalmologists have embraced the advantages that this new technology provides. However, there is a gap in the current literature concerning the patient perspective on the burden of their wet AMD monitoring, particularly during the confinement due to COVID-19, and telemedicine. Therefore, there is a need to understand the patients’ perspectives regarding the future use of telemedicine in the monitoring of their wet AMD.

Our study aimed to present the patients’ subjective opinions concerning the effect of the COVID-19 pandemic on the monitoring of their wet AMD, their attendance of their scheduled intravitreal anti-VEGF injection visits during the first period of the national confinement due to COVID-19 in Switzerland, as well as the possibility of using telemedical services in our clinic in future similar circumstances.

## 2. Materials and Methods

Study participants: This study was conducted in Swiss Visio Montchoisi and RétinElysée Ophthalmology Centre in Lausanne, Switzerland. This study adhered to the Declaration of Helsinki [17] and its amendments and was approved by the Swiss Ethics Committee (approval number: 2020-01912). Patients diagnosed with nAMD of any type (I, II, or III), that had received intravitreal anti-VEGF injections both before and after the first period of COVID-19 confinement in Switzerland (13 March 2020–27 April 2020), regardless of adherence to the injection schedule, were recruited when they visited the clinics. Patients with nAMD of any type (I, II, or III) that underwent intravitreal anti-VEGF injections both before and after the period of confinement due to the COVID-19 pandemic, were included in this study after providing informed consent. Patients who underwent intravitreal anti-VEGF injections for different retinal pathologies than nAMD (e.g., central retinal vein occlusion, branch retinal vein occlusion) were excluded from the study. The patient recruitment and data collection were performed from September 2020 until June 2021. Each patient was asked to complete two questionnaires.

The first questionnaire evaluated the subjective views of the patients on their adherence to their scheduled intravitreal anti-VEGF injections during the period of confinement, and the possibilities of the applications of telemedicine in the years to come. The other questionnaire was a visual function questionnaire adapted from the National Eye Institute Visual Function Questionnaire-25 (NEI-VFQ25) [18] which focused on patients’ opinions concerning their visual function change during the first period of COVID-19 confinement in Switzerland. The questions captured in both questionnaires were yes/no questions, or multiple-choice questions. Both questionnaires are available.

Each patient was given thorough instructions regarding the completion of the questionnaires. Each participant filled their questionnaires having no time restrictions either at their home or in the clinic, or on their own or assisted by their family members. Concerning the future use of telemedicine in the management of their wet AMD, our research team thoroughly explained to the patients that there are certain BCVA and OCT features that predict the course of their wet AMD, as well as their individualized intravitreal anti-VEGF injection treatment. We also described to them the basic characteristics of the telemedicine model that could be used in our clinic: BCVA, intraocular pressure, colour fundus photography, and macula OCT would be incorporated in our telemedicine model. Those measurements could be performed by a trained technician in a telemedical centre or remotely by the patients themselves under the guidance of their ophthalmologist. The data would be stored on a database, which would then be reviewed electronically by their ophthalmologist. The ophthalmologist would then decide which patients had threatening OCT features and were in greater risk of wet AMD progression, and therefore were in need of immediate intravitreal anti-VEGF injection.

All patients provided written informed consent to participate in this study. The patients were divided in two groups: group 1 included the patients that declared non-adherence to their assigned injection interval during the first period of COVID-19 confinement, whereas group 2 included the patients that responded that they adhered to it.

Statistical analysis: The SAS software (SAS 9.1, SAS Institute Inc., Cary, NC, USA) was used to perform the statistical analysis. Descriptive and inferential statistics were performed using the questions of the NEI-VFQ25 [18] questionnaires. The variables tested were categorical variables and numeric and ordinal score variables. Mean score values and frequencies were expressed with their standard deviation (±SD) and percentages (%), respectively. Rank Welch test and Wilcoxon rank sum test were used for the comparison of variables between groups. Power analysis for a Z-test (two proportions, two-sided) was conducted, and a sample size of ~180 observations was chosen to detect a difference of 0.2 with an alpha set to 5% and a power of 80%. The statistical significance level was set at two-sided a = 0.05.

## 3. Results

Out of the 185 eligible patients, 130 (70.3%) responded to the questionnaires. The average age was 80.02 ± 9.81 years. Out of the 130 patients that responded to the questionnaires, 91 patients (70.0% of total) were female, and 39 patients (30.0% of total) were male. The patients of group 1 (11 patients, 8.5% of total) declared non-adherence to the injection interval that was assigned to them during the first period of COVID-19 confinement, whereas the patients of group 2 (119 patients, 91.5% of total) responded that they did.

Among patients in group 1, 37.5% declared non-attendance to their scheduled injection visit because they preferred not to use public transportation, 25.0% based on their family’s advice, and 25.0% because they were not reminded of their scheduled visit or were unaware of the clinic’s availability. In contrast, 78.8% of patients in group 2 considered the treatment continuation to be of higher importance than the risk of COVID-19 contraction, 43.7% were not afraid of contracting it, and 42.9% used private transportation to ensure their safety.

The majority of patients in this study (81.8% of group 1 and 81.9% of group 2) were not familiar with telemedicine, without any significant difference between both groups (*p* = 0.99).

In the pre-lockdown period, patients of group 2 were significantly more likely to rely on help from others than the patients in group 1 (mean NEI-VFQ25 [18] score: 4.17 ± 1.25 vs. 4.64 ± 0.50, *p* = 0.02). The patients in group 2 had significantly more difficulty reading traffic or store signs on the street (mean NEI-VFQ25 [18] score:1.90 ± 1.14 vs. 1.36 ± 0.50, *p* = 0.009), walking down steps, stairs, or curbs at night or in poor lighting (mean NEI-VFQ25 [18] score: 2.39 ± 1.13 vs. 1.64 ± 0.67, *p* = 0.005), noticing what was on the side when they walked (mean NEI-VFQ25 [18] score: 1.65 ± 0.90 vs. 1.09 ± 0.30, *p* < 0.0001), seeing people’s reactions (mean NEI-VFQ25 [18] score: 1.48 ± 1.03 vs. 1.09 ± 0.30, *p* = 0.005), and choosing and matching their clothes (mean NEI-VFQ25 [18] score: 1.36 ± 0.90 vs. 1.09 ± 0.30, *p* = 0.04) than the patients in group 1. Finally, patients in group 2 declared to be more worried about their vision than the patients in group 1 (mean NEI-VFQ25 [18] score: 2.80 ± 1.01 vs. 2.27 ± 0.79, *p* = 0.06).

In the period of lockdown, the NEI-VFQ25-adapted visual function of the patients in group 1 was not significantly different than that of patients in group 2.

Finally, in the possibility of a lockdown in the years to come, 36.3% of group 1 and 8.7% of group 2 would rather monitor their wet AMD via telemedicine (*p* = 0.02), 54.5% and 86.9% would rather visit the clinic (*p* = 0.02), while 9.0% and 4.3% would avoid the visit, respectively. The majority of patients in group 1 (70.0%) and one-third of patients in group 2 (33.6%) would prefer to use the telemedicine application at home than to visit a telemedicine centre, either by themselves or through help from a family member or non-medical technical staff (*p* = 0.04).

## 4. Discussion

Our study evaluated the patients’ perspective on the monitoring of their wet AMD during COVID-19 and the importance of telemedicine and found that, in the event of future COVID-19 confinement or any similar circumstances, 36.3% of patients in group 1 and 8.7% of patients in group 2 would rather monitor their wet AMD through telemedicine, 54.5% and 86.9% would rather monitor it in the clinic, while 9.0% and 4.3% would cancel the appointment to the clinic, respectively.

The abovementioned findings showed that the patients who did not abide by their scheduled intravitreal anti-VEGF injection visits during the confinement were more likely to favour the use of telemedicine to monitor their wet AMD. In comparison, the patients who abided by their injection intervals were more likely to prefer visiting the clinic. Moreover, the patients that did not abide by the schedule were more than twice as likely to avoid the visit. When the patients were asked about their preference regarding telemedicine use for wet AMD monitoring, those in group 1 were more likely to use the application at home, by themselves, or with the help of a family member or non-medical staff, whereas patients in group 2 were more likely to use the application in a telemedicine facility near their home.

The findings of this study also indicate that the patients in group 1 were either more afraid of contracting COVID-19 or that their visual function was not as severely impaired. Interestingly, in the pre-lockdown period, the patients of group 2 declared to be more likely to be visually impaired, as they relied on more help from others, and they were less functional in everyday activities.

Another important finding that should be mentioned is that in our study, group 1 only represents 8.5% of the patients that responded to the questionnaires, whereas group 2 represents 91.5%. As previously mentioned, the groups represent the patients’ opinions concerning their adherence to their scheduled intravitreal anti-VEGF injection visits. We retrospectively analysed the same cohort of patients regarding their BCVA and optical coherence tomography changes between their first post- and last pre-confinement visit in our study titled “The importance of monitoring wet age-related macular degeneration patients during Coronavirus disease 19 pandemic: A retrospective study of assessment of functional and structural outcomes” [19]. The actual percentage of each group differed drastically from the abovementioned percentages, as the patients that missed at least one of their scheduled injection visits during the period of confinement actually represented 48.1% of the total, whereas those who kept their injection interval during the same period actually represented 51.9% of the total. Therefore, it is clear that many patients believe that they respected their intervals when in reality, they did not. This discrepancy may be due to two main reasons. To begin with, the first period of Swiss COVID-19 confinement resulted in a major and abrupt alteration in everyone’s daily routines, including our patients’. Many of them may have forgotten whether or not they actually attended their scheduled visit, and hence indicated that they did. Second, although our study team instructed them to answer the surveys objectively, many patients show a bias when filling out the surveys and tend to choose the ideal responses rather than the genuine ones.

It is rather interesting that 37.5% of patients in group 1 preferred to miss their planned injection because they did not want to use public transportation and that another 25.0% of the same group did not attend because their families advised against it. Given the fact that the actual number of patients in group 1 is much higher than that indicated by the patients themselves, the fear of contracting COVID-19 seems to be of paramount importance for these patients and their families. This may be due to the fact that during the first national lockdown in Switzerland, there was much more fear and uncertainty towards the pandemic and more people were afraid to leave their homes than they were in the period after.

The first limitation of the study is the fact that many patients responded that they attended their scheduled intravitreal anti-VEGF injection visit during the first period of Swiss confinement, although they did not. This is an inherent limitation, as the objective of our study was to evaluate the patients’ perspective concerning their injection adherence during the COVID-19 confinement and the importance of telemedicine, therefore subjectivity could not be avoided. Second, although the patients were questioned about the possibility of monitoring their wet AMD via telemedicine in the future, they had no prior experience with this technology and so could not assess its utility in monitoring their wet AMD in a practical manner.

The pandemic of COVID-19 increased the awareness regarding distance medicine with the use of artificial intelligence in the screening and management of wet AMD [20,21].

The pandemic of COVID-19 that led to the national confinement in Switzerland created a unique circumstance in which a significant percentage of wet AMD patients failed to receive their scheduled treatment, despite the availability of our clinics to perform continuous intravitreal anti-VEGF injections. The results of this study suggest that in the event of another confinement due to COVID-19 or any other similar circumstances, the biggest part of the patients would still rather visit the clinic. As for the patients who did not receive their intravitreal anti-VEGF injections during the first period of the national confinement due to COVID-19 in Switzerland, they were more likely to prefer remote monitoring through telemedicine than the patients that abided by their planned intravitreal anti-VEGF injections during that period.

## 5. Conclusions

During circumstances similar to the COVID-19 confinement, the majority of the patients would prefer a visit to the clinic. The patients that declared non-adherence to their assigned injection interval during the first period of COVID-19 confinement (group 1) would prefer to monitor their wet AMD via telemedicine at a higher rate than the group of the patients that responded that they adhered to their assigned injection interval during the first period of COVID-19 confinement (group 2). More prospective studies evaluating patients’ opinions on telemedicine monitoring of their wet AMD are needed, with patients having the opportunity to use the telemedicine applications for two or three months to do an objective assessment.

## Data Availability

The datasets used and/or analysed during the current study are available from the corresponding author on reasonable request.

## Abbreviations

AMD	age-related macular degeneration
anti-VEGF	intravitreal anti-vascular endothelial growth factor
BCVA	best-corrected visual acuity
COVID-19	coronavirus disease 2019
nAMD	neovascular AMD
NEI-VFQ25	National Eye Institute Visual Function Questionnaire-25
OCT	optical coherence tomography
SD	standard deviation
